# Prize Medals of the Apothecaries' Company and the College of Surgeons

**Published:** 1837-01

**Authors:** 


### BOTANICAL PRIZE MEDALS OF THE APOTHECARIES' COMPANY.

The gold medal has been this year granted to Mr. Jenner, and the silver medal to Mr. Tegetmeir. Both these gentlemen are, we believe, pupils of the London University.

---

### THE JACKSONIAN PRIZE OF THE COLLEGE OF SURGEONS.

The subject for 1837 is " An Inquiry into the Nature of the Processes of Suppuration and Ulceration." The Dissertations are to be addressed to the Secretary, and delivered before Christmas-day, 1837.

